# Successful Retrieval of Human Papillomavirus DNA in Veil-Based Collected Female Genital Secretions After Long-Term Storage in Universal Transport Medium

**DOI:** 10.3390/diagnostics15091079

**Published:** 2025-04-24

**Authors:** Jonathan Muwonga Tukisadila, Juval Avala Ntsigouaye, Serge Tonen-Wolyec, Ralph-Sydney Mboumba Bouassa, Jeremie Muwonga, Laurent Belec

**Affiliations:** 1 École Doctorale Régionale D’Afrique Centrale en Infectiologie Tropicale, Franceville 876, Gabon; juvalavala@gmail.com (J.A.N.); wolyec@gmail.com (S.T.-W.); ralphsmbouassa@montfort.on.ca (R.-S.M.B.); 2 Laboratoire de Biologie Clinique des Cliniques Universitaires de Kinshasa, Kinshasa 123, Democratic Republic of the Congo; pmuwonga@hotmail.com; 3 Laboratory of Virology, Hôpital Européen Georges Pompidou, Assistance Publique-Hôpitaux de Paris (AP-HP), 75015 Paris, France; laurent.belec@aphp.fr; 4 Department of Internal Medicine, Faculté de Médecine, Université de Bunia, Bunia 292, Democratic Republic of the Congo; 5 Department of Internal Medicine, Faculté de Médecine et Pharmacie, Université de Kisangani, Kisangani 2012, Democratic Republic of the Congo; 6 Institut du Savoir Montfort, Montfort Hospital, Ottawa, ON K1K 0T2, Canada; 7 Department of Family Medicine, Faculty of Medicine, University of Ottawa, Ottawa, ON K1N 6S1, Canada; 8 Faculté de Médecine Paris Descartes, Université Paris Cité, 75006 Paris, France

**Keywords:** human papillomavirus (HPV) DNA preservation, self-sampling-based HPV screening, genital veil, multiplex real-time PCR, HPV viral load, biobanking, universal transport medium, sub-Saharan Africa

## Abstract

**Background/Objectives**: The surveillance of viral strain evolution is needed during prophylactic HPV vaccination programs against cervical cancer and necessitates safely archiving and storing cervical samples while maintaining the long-term stability of HPV DNA to carry out molecular diagnosis. The present proof-of-concept study aimed to assess DNA stability for HPV molecular detection from veils resuspended in a universal transport medium (UTM) and conserved at different temperatures after long-term storage. **Methods**: The detection and quantification of HPV DNA were evaluated in female genital secretions self-collected using veils and conserved in Cyt-All^®^ UTM at −30 °C, +4 °C, and +25 °C after long-term 27-month storage. **Results**: A slight degradation of the ubiquitous single-copy cellular DNA TOP3 gene was assessed using multiplex real-time PCR (BMRT Human Papillomavirus Genotyping Real Time PCR Kit, Bioperfectus Technologies Co., Ltd., Taizhou, Jiangsu, China) at positive temperatures (+4 °C and +25 °C) but not at a frozen temperature (−30 °C) after 27 months of storage. Nevertheless, HPV DNA preservation was sufficient at the three storage temperatures to detect and quantify HPV DNA, with a similar rate of HPV detection, a similar level of cumulative HPV viral loads, high sensitivity and specificity, and perfect concordance in HPV genotype detection after the long period of 27 months of storage. Finally, the conservation of genital samples for a prolonged period in the Cyt-All^®^ medium, even at room temperature, allows for the detection and quantification of any HPV and HR-HPV with high accuracy. **Conclusions**: The combination of veil-based self-sampling of female genital secretions and their elution and conservation in UTM may be used in the field to carry out longitudinal molecular epidemiology surveys of circulating HPV.

## 1. Introduction

Cervical cancer associated with oncogenic high-risk human papillomavirus (HR-HPV) remains the second leading cause of cancer-related deaths in sub-Saharan Africa [1,2,3,4,5]. The estimates for Africa show that 125,699 new cases of cervical cancer and 80,614 associated deaths were recorded in 2022 [5].

The World Health Organization (WHO) recently called for the elimination of cervical cancer thanks to vaccination, screening, and treatment [6]. Knowledge on HPV prevalence and HR-HPV genotype distribution is essential for the implementation of effective prophylactic vaccination programs and appropriate epidemiological monitoring of viral ecology before and after vaccination in specific sub-Saharan African geographical settings and populations [1,7]. Continued surveillance of HPV infections and related precancerous and cancerous lesions of the cervix is still required to assess, at the population level, the effectiveness of prevention strategies against cervical cancer. Regarding HPV infections, there is a particular interest in monitoring HPV genotypes among vaccinated and unvaccinated females to track potential HPV vaccine failure and/or HPV type replacement [8,9]. Therefore, there is a need to safely archive and store cervical samples while maintaining the long-term stability of HPV DNA to carry out molecular diagnoses.

The vaginal veil is listed as a sampling device for HPV tests designed for self-collection by UNITAID [10]. We previously showed that the self-sampling of female genital secretions using veils constitutes a highly appreciated and useful tool to collect genital samples for molecular analysis, especially HPV molecular detection [11]. Thus, Nodjikouambaye et al. reported that the rates of any HPV DNA and HR-HPV DNA positivity in African women were significantly higher (1.67- and 1.57-fold, respectively; *p* < 0.0001) when using genital secretions collected by veil (Vaginal Veil Collector V-Veil UP2™ device, V-Veil-Up Production SRL, Arges, Romania) than clinician-collected cervical secretions by swab [11]. Using the same veil medical device, the Vitroveil^®^ (VITRO SA, Granada, Spain) self-sampling device enables Spanish women to obtain a higher prevalence of molecular detection of any HR-HPV than clinician-collected samples by cervical scraping (30.6% versus 24.3%; *p* < 0.0001) [12]. Several elution liquids can be used to extract DNA from a veil impregnated with female genital secretions with excellent yields [11,12].

Furthermore, cervical cancer ranks as both the second most frequent and most deadly female cancer in the Democratic Republic of the Congo (DRC) [5], where around one-quarter of adult women were found to harbor HR-HPV in their genital secretions in the capital city Kinshasa [13]. We recently used the Vaginal Veil Collector V-Veil UP2™ device (V-Veil-Up Production SRL) with UTM (Cyt-All^®^, Alphapath, Mauguio, France) as the elution liquid to conduct a descriptive cross-sectional study on genital HPV and HR-HPV carriage in female sex workers (FSWs) living in eastern DRC, whose preliminary results were presented elsewhere [14]. UTM is designed to preserve a wide range of viral and bacterial organisms. This versatility makes it suitable for various diagnostic applications, simplifying the collection process. UTM is formulated to maintain the integrity of viral samples during transport, ensuring accurate test results. This is vital because viruses are fragile and can degrade quickly if not stored properly. Furthermore, UTM has to be compatible with modern molecular diagnostic techniques in order to allow for the sensitive and specific detection of viral nucleic acids. Finally, it helps to stabilize samples during transport, which is especially important when specimens need to be sent to distant laboratories.

This survey also enabled us to assess stability over a prolonged period for DNA extracted from veils impregnated with genital secretions. Thus, the present proof-of-concept study aimed to assess DNA stability for HPV molecular detection from veils resuspended in UTM and conserved at different temperatures after 27 months of storage.

## 2. Materials and Methods

### 2.1. Study Design, Population, and Sample Processing

In 2022, a descriptive cross-sectional study including 415 consenting and consecutive FSWs (mean age: 28.1 years; range: 18–59) living in Kisangani, the capital of the Tshopo province in the DRC, was conducted on genital HPV, HR-HPV, possibly oncogenic HPV (PO-HPV), and low-risk HPV (LR-HPV). In this province of the DRC, the prophylactic vaccination program against oncogenic HPV has only just begun and is still very limited. At inclusion, no participants had been vaccinated with the HPV prophylactic vaccine. Genital sampling was carried out through the genital self-collection method with veils (Vaginal Veil Collector V-Veil UP2™ device, V-Veil-Up Production SRL), as previously described [11]. The Vaginal Veil Collector V-Veil UP2™ consists of non-woven hydrophilic polyethylene [11]. The vaginal veil safely and gently catches and retains the female genital secretions, thus harvesting cells, proteins, and nucleic acids (DNA/RNA). Like what was reported for polyethylene non-woven polyesters [15], through its composition, the veil device does not absorb the liquids, but rather adsorb the genital’s biochemical components, thereby allowing for their full desorption after adequate elution of the collected biological material. Along with its special biomaterial composition, what makes the veil so original is its large external surface [11], allowing it to easily adsorb the maximum amount of proteins and nucleic acids and further release them, efficiently, during the elution phase according to the established concentration gradient.

The impregnated veils were placed in a 15 mL plastic conical tube and kept frozen at −30 °C. The veils were then transported in dry ice to the virology laboratory of the hôpital européen Georges Pompidou, (English equivalent: Georges Pompidou European Hospital), Paris, France, and kept frozen at −30 °C.

For the molecular detection of HPV, the frozen veils were placed in 10 mL of the Cyt-All^®^ transport medium. This preservation medium contains no formaldehyde or methanol but has ethanol and is thus flammable (H225). According to the instructions for use (https://www.alphapath.fr/cyt-all/ accessed on 9 April 2025), the Cyt-All^®^ liquid allows stability for molecular analysis for at least 82 days at room temperature (+2 °C to +32 °C).

All genital samples were tested using the Papilloplex^®^ HR-HPV DNA Kit and the Papilloplex^®^ LR-HPV Kit (GeneFirst Ltd., Abingdon, UK), as shown in the VALGENT framework-validated reference HPV assays for HPV genotyping [16].

For the stability study, 30 samples of eluted veils conserved in the Cyt-All^®^ medium were selected at random from those found to be HR-HPV-positive after HPV molecular detection with reference assays as well as 30 other samples found negative for any HPV as negative controls. After vortexing for 1 min to thoroughly homogenize the eluted secretions, three aliquots of 1 mL of each selected sample were prepared for storage at −30 °C, +4 °C (common refrigerator temperature), and +25 °C (considered as “average room temperature” in sub-Saharan Africa, like in the DRC) until further processing. The storage period was arbitrarily set at 27 months, i.e., 10 times the duration period recommended by the manufacturer.

### 2.2. DNA Extraction for the Stability Study

DNA was further extracted from 200 µL of cervicovaginal secretions eluted in the Cyt-All^®^ medium using the QIAamp^®^ DNA Mini Kit (Qiagen, Hilden, Germany) according to manufacturer’s instructions, and the DNA was eluted in 100 µL of the kit’s elution buffer before genotyping. The extracted DNA was stored at −30 °C until analysis. DNA was quantified using a Qubit^®^ dsDNA BR Assay kit with the Qubit 2.0 fluorimeter (Thermo Fisher Scientific Inc., Waltham, MA, USA).

### 2.3. HPV Genotyping and Quantitation and Ubiquitous TOP3 Cellular Gene Detection via Multiplex PCR

A total of 1 µg of extracted DNA was further used for PCR analysis. HPV detection and genotyping were carried out using the fluorescence-based Bioperfectus Multiplex Real-Time (BMRT) Human Papillomavirus Genotyping Real-Time PCR Kit (Jiangsu Bioperfectus Technologies Co., Ltd., Taizhou, Jiangsu Province, China). The BMRT HPV kit contains primers and the corresponding TaqMan probes that amplify a 100-base-pair L1 amplicon, as previously described [17]. According to the HPV classification nomenclature provided by the International Agency for Research on Cancer (IARC) [18], the BMRT HPV Genotyping Real-Time PCR Kit allows us to distinguish each of the 21 most prevalent HPV genotypes, including 13 HR-HPV (HPV-16, -18, -31, -33, -35, -39, -45, -51, -52, -56, -58, -59, and -68), 5 PO-HPV (HPV-26, -53, -66, -73, and -82), and 3 LR-HPV (HPV-6, -11, and -81). The BMRT HPV kit was used according to the manufacturer’s instructions. Briefly, eight reactions per sample were performed simultaneously. Among them, reactions A, B, C, D, E, F, and G were prepared to detect and differentiate, in FAM™/VIC^®^ (HEX)/ROX™ fluorescent channels, HPV-16/-18/-31, HPV-59/-66/-53, HPV-33/-58/-45, HPV-56/-52/-35, HPV-68/-51/-39, HPV-73/-26/-82, and HPV-6/-11/-81, respectively. In addition, an internal control (IC) with the housekeeping single-copy human gene TOP3, which encodes the human DNA topoisomerase III enzyme [19], in reaction tube H (FAM™ channel) was set to simultaneously identify possible PCR inhibition and confirm the reliability of the reagents in this kit as well as the viral loads. PCR was performed using the CFX96 real-time PCR instrument (Bio-Rad, Marnes-la-Coquette, France).

To validate the PCR reaction, the cycle threshold (Ct) values of positive controls had to be less than or equal to the cut-off value of 30.0 in FAM™, VIC^®^ (HEX) and ROX™, and the blank controls had to be undetectable. The optical unit of the real-time PCR system measured the emitted fluorescence. For each of the 21 detected HPV genotypes, the qualitative reference values of the positive cut-off were assessed by the manufacturer using ROC curves based on clinical trial results. Specimens with Ct values less than or equal to the cut-off value of one given HPV type were considered as positive for this HPV genotype. The cut-off values for Ct were those provided by the manufacturer in the instructions for use, ranging for positivity from 34.6 to 36.9 in FAM™, from 35.5 to 37.0 in VIC^®^ (HEX), and from 34.5 to 35.6 in ROX™. Conversely, specimens with Ct values above the cut-off value of one given HPV type were considered as negative.

The Perfectus HPV Analyzer Software v1.0 (Jiangsu Bioperfectus Technologies Co., Ltd., Taizhou, Jiangsu Province, China) was applied for the quantitative viral loads of the 21 detected HPV.

### 2.4. Statistical Analysis

The data were compiled on an Excel file and analyzed using GraphPad Prism version 8.4.2 (GraphPad Software, Inc., San Diego, CA, USA). Means and standard deviations were calculated for quantitative variables. The HPV DNA test results with the Papilloplex^®^ assays (GeneFirst Ltd.) were used as the reference standard to estimate the sensitivity, specificity, agreement, and accuracy between immediate versus delayed methods to detect HPV. The overall prevalences of HPV DNA detection (any genotypes, HR-HPV, PO-HPV, LR-HPV, and HPV genotypes targeted by the Gardasil-9^®^ vaccine (Merck & Co. Inc., Rahway, NJ, USA)) between immediate versus delayed methods to detect HPV were compared using the McNemar’s test for paired data. The agreement between immediate versus delayed methods to detect HPV was estimated using Cohen’s κ coefficient [20], and the degree of agreement was determined as ranked by Landlis and Koch [21]. Percent agreement corresponded to the observed proportion of identical results between immediate versus delayed methods to detect HPV. The Youden J index was used to determine the accuracy of storage conditions (Cyt-All^®^ UTM and temperatures) to allow for a correct diagnosis of HPV [22]. A *p*-value of <0.05 was considered statistically significant.

## 3. Results

### 3.1. Selected Study Population

**Figure 1:**
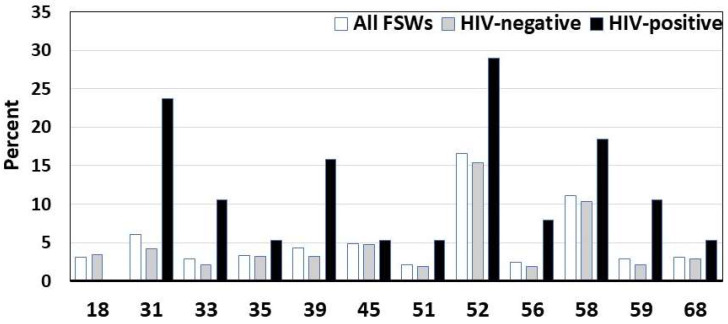
Distribution by HIV status of high-risk HPV (HR-HPV) in veil-based collected genital samples positive for HPV molecular detection using the Papilloplex^®^ HR-HPV DNA Kit (GeneFirst Ltd., Abingdon, UK) among 415 adult female sex workers living in Kisangani, Democratic Republic of Congo.

The overall HIV prevalence was 9.1% [95% confidence interval (CI): 6.3–11.9%]. The overall prevalences of any HPV and HR-HPV infections detected using the reference Papilloplex^®^ assays were 92.7% and 36.9%, respectively. The overall prevalence of HR-HPV was 36.9% [95% CI: 32.3–41.5%] and was higher in HIV-positive than uninfected FSWs (*p* < 0.001) (Figure 1). The most prevalent HR-HPVs detected were HPV-52 (16.6%) and HPV-58 (11.1%). HPV-16, HPV-18, and HPV-45 were poorly represented in only 6.7%, 3.1%, and 4.8% of FSWs, respectively. Therefore, 63.4% of HR-HPV would be covered by the Gardasil-9^®^ vaccine.

The mean age of the 30 selected HPV-positive FSWs was 25.7 years (range: 18–37), and that of the 30 HPV-negative controls was 36.9 years (range: 23–44).

### 3.2. HPV Detection and Genotypes After 27 Months of Storage

There was no significant difference in the yield of DNA measured after storage at all storage temperatures. All samples from HPV-negative controls were negative at all storage temperatures according to the BMRT Human Papillomavirus Genotyping Real-Time PCR Kit.

Table 1 depicts the genotypes of the HPV detected at the three storage temperatures among the 30 selected HPV-positive FSWs. At storage temperatures of −30 °C, +4 °C, and +25 °C, 100, 98, and 95 different HPVs, respectively, were detected in this study’s participants using the BMRT Human Papillomavirus Genotyping Real-Time PCR Kit. At the reference storage temperature of −30 °C, the most frequently detected HPV genotypes were both the HR-HPV-52 and the potentially oncogenic HPV-53 (14.0%), followed by the potentially oncogenic HPV-66 (11.0%) and HR-HPV-58 (10.0%). This suggested a good representativeness of the randomization. In addition, 69.7% of HR-HPV detected would be covered by the Gardasil-9^®^ vaccine. The prevalences of HPV DNA detection and the HPV genotypes detected were similar at the three storage temperatures.

### 3.3. HPV Viral Load and TOP3 Gene Detection After 27 Months of Storage

**Figure 2:**
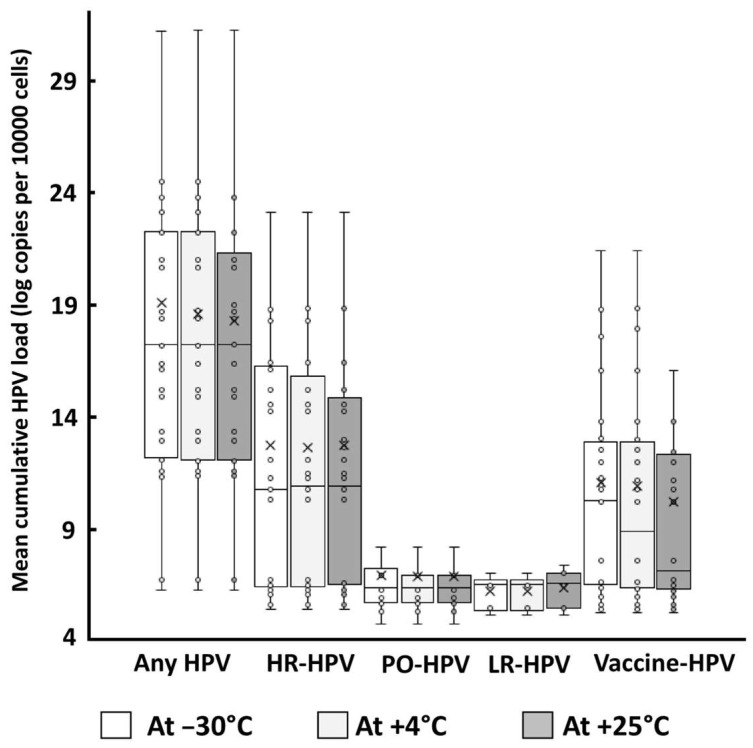
Box plots of the cumulative HPV viral load of any HPV, HR-HPV, PO-HPV, LR-HPV, and Gardasil-9^®^ vaccine HPV, expressed in log copies per 10,000 cells, as assessed using the BMRT Human Papillomavirus Genotyping Real-Time PCR Kit (Jiangsu Bioperfectus Technologies Co., Ltd.), in 30 veil-based self-collected genital samples positive at inclusion, which were determined using Papilloplex^®^ High-Risk HPV and Papilloplex^®^ Low-Risk HPV (GeneFirst Ltd.) as the reference assays, and samples were stored in the Cyt-All^®^ UTM (Alphapath) at −30 °C, at +4 °C, and at room temperature (+25 °C) for a 27-month period. Each box is drawn from the first quartile to the third quartile, with a horizontal line drawn inside it to denote the median.

Figure 2 depicts the mean cumulative HPV viral loads expressed in log copies per 10,000 cells of the HPV detected at the three storage temperatures, including any HPV, HR-HPV, PO-HPV, LR-HPV, as well as the HPV types covered by the 9-valent Gardasil-9^®^ vaccine, without significant differences between groups and storage temperatures (any HPV: 18.8 ± 9.7 at −30 °C, 18.3 ± 8.8 at +4 °C, and 18.0 ± 8.7 at +25 °C; HR-HPV: 12.5 ± 8.9 at −30 °C, 12.4 ± 8.5 at +4 °C, and 12.4 ± 9.0 at +25 °C; PO-HPV: 6.7 ± 1.9 at −30 °C, 6.6 ± 1.8 at +4 °C, and 6.5 ± 2.1 at +25 °C; LR-HPV: 5.9 ± 0.7 at −30 °C, 5.8 ± 0.8 at +4 °C, and 6.1 ± 0.8 at +25 °C; and vaccine-targeted HPV: 10.8 ± 6.8 at −30 °C, 10.6 ± 7.0 at +4 °C, and 9.5 ± 6.5 at +25 °C; without statistical differences for all comparisons; *p* > 0.05).

**Figure 3:**
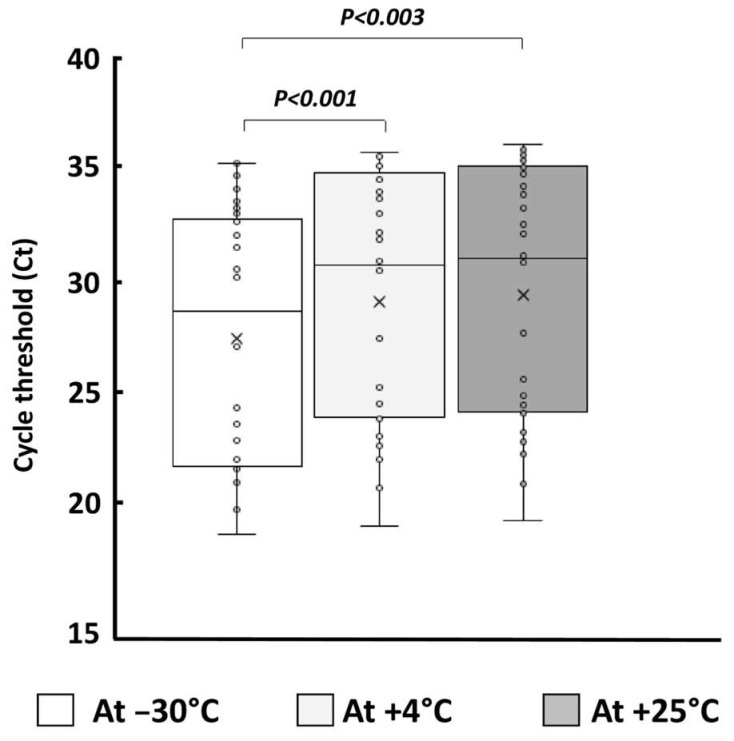
Box plots of the estimated quantity of the ubiquitous single-copy human gene TOP3 encoding DNA topoisomerase III, expressed in cycle threshold (Ct) arbitrary units, as assessed by the BMRT Human Papillomavirus Genotyping Real-Time PCR Kit (Jiangsu Bioperfectus Technologies Co., Ltd.), in 30 veil-based self-collected genital samples positive at inclusion, which were determined using Papilloplex^®^ High-Risk HPV and Papilloplex^®^ Low-Risk HPV (GeneFirst Ltd.) as the reference assays, and samples were stored in the Cyt-All^®^ UTM (Alphapath) at −30 °C, at +4 °C, and at room temperature (+25 °C) for a 27-month period. Each box is drawn from the first quartile to third quartile, with a horizontal line drawn inside it to denote the median.

The ubiquitous TOP3 gene was detected in all samples. Figure 3 shows the estimated quantity of the TOP3 gene, as assessed by Ct in arbitrary units at the three storage temperatures. The mean Ct at +4 °C (29.0 ± 5.4) and +25 °C (29.3 ± 5.4) was higher than that at −30 °C (27.4 ± 5.4) (*p* < 0.001 and *p* < 0.003, respectively).

### 3.4. Agreement Between Immediate HPV Detection Using Reference Molecular Assays and Delayed Detection After 27 Months of Storage

Delayed detection of any HPV and HR-HPV in the veil-based self-collected genital samples stored in the Cyt-All^®^ UTM using the BMRT Human Papillomavirus Genotyping Real-Time PCR Kit showed high sensitivity and accuracy at all storage temperatures, ranging from 92.2% to 97.1% for any HPV and from 89.4% to 96.9% for HR-HPV (Table 2).

The percent agreements between immediate versus delayed methods to detect any HPV and HR-HPV ranged from 93.9% to 97.7% and from 92.7% to 97.9%, respectively. Cohen’s κ coefficients were between 0.84 and 0.94 for any HPV and from 0.84 to 0.95 for HR-HPV, demonstrating “almost perfect” agreement between immediate versus delayed methods to detect any HPV and HR-HPV [21] (Table 2).

## 4. Discussion

Primary (prophylactic vaccination) and secondary (molecular screening) prevention strategies form the basis of cervical cancer elimination worldwide [1]. In this context, epidemiological surveillance of circulating HPV remains crucial to assess the efficacy and impact of screening and vaccination policies [6]. Thus, the growing use of HPV molecular testing in cervical cancer screening enhances the opportunity to use self-collected genital secretions as an innovative approach to improve coverage rates [1,11,23,24,25]. Accordingly, the validation and standardization of preanalytical procedures are essential for the quality assurance of HPV tests on self-collected samples. In practice, there is a need to safely store genital samples to conduct long-term studies in vaccinated and non-vaccinated subjects.

The overall prevalences of any HPV and HR-HPV detection in this study’s participants were 92.7% and 35.4%, respectively. In a recent meta-analysis of sub-Saharan African women visiting health facilities for various gynecological problems, the pooled prevalence of HR-HPV infection was estimated at 34.0% (95% CI: 29.0–39.0%) [26], a rate similar to the HR-HPV prevalence in this study’s FSWs. The distribution of HPV in genital samples in included FSWs was atypical, specific, and unique, with high rates of HR-HPV genotypes detected in one-third, with HR-HPV-52 (14.9%) and HR-HPV-58 (10.1%) being the predominant genotypes. These observations suggest that the signature of the most common HPV genotypes detected in FSWs living in Kisangani comprises HPV types generally observed in FSWs in the world, whereby the distributions of HPV-52 (7.9%) and HPV-58 (5.6%) are elevated [27].

This study aimed to evaluate the possibility of detecting and quantifying HPV DNA in female genital secretions self-collected using veils and conserved in the UTM at various temperatures after a long-term 27-month storage period. In our study, the quality of the DNA was assessed by evaluating the housekeeping TOP3 gene in all samples, which were all found to be adequate. Interestingly, the Bioperfectus HPV assay relies on the amplification of only 100 bp fragments, a feature that could be associated with better PCR efficiency, such as when using long-term DNA extracted from genital specimens. Despite that, the ubiquitous TOP3 gene was detected in all samples. We herein provide evidence for a slight degradation of the ubiquitous cellular DNA TOP3 gene, as assessed by a significant increase in the Ct gene value via multiplex real-time PCR, at positive temperatures (+4 °C and +25 °C) but likely not at the frozen temperature (−30 °C) after 27 months of specimen storage. Nevertheless, HPV DNA preservation was sufficient at the three storage temperatures to detect and quantify HPV DNA using BMRT multiplex quantitative PCR, with similar rates of HPV detection, similar levels of cumulative HPV viral loads, high sensitivity and specificity, and almost perfect concordance in HPV genotype detection after the long period of 27 months of storage. Finally, the conservation of genital samples for a prolonged period in UTM, even at room temperature, allows us to detect and quantitate any HPV and HR-HPV with high accuracy, including HPV genotypes targeted by the Gardasil-9^®^ vaccine. Thus, the slight degradation of cellular DNA observed over time contrasts with the resistance of HPV DNA, likely because the viral genome is surrounded by a highly resistant icosahedral nucleocapsid, which renders HPV extremely steady in various environments [28,29]. Taken together, these findings demonstrate that veil-based self-collected female genital secretions eluted and conserved in the UTM for a prolonged period of at least 27 months, such as those stored in a freezer (−30 °C), in a refrigerator (+4 °C), or at room temperature, constitute a convenient matrix to detect and quantitate HPV DNA through multiplex PCR. The combination of self-sampling of female genital secretions and their elution and conservation in the UTM, such as the Cyt-All^®^ UTM, may be used to carry out longitudinal molecular epidemiology surveys of circulating HPV in the field in order to monitor the evolution of viral strains during prophylactic vaccination programs against cervical cancer.

Our observations are reminiscent to previous reports that show the stability of HPV DNA conserved in several viral preservative media and in various experimental and storage conditions, such as over time ranging from several weeks [30,31,32] to several months [33,34,35,36,37], up to 2.5-years of storage in BD SurePath and Hologic PreservCyt liquid-based cytology media at +2–8 °C [34], and up to 4.5 years on a Flinders Technology Associates (FTA) matrix card at room temperature [38], with the possibility of effective molecular analysis after the period of storage. The long-term possibility of HPV DNA molecular analysis from self-collected female genital samples with different vaginal collection devices was previously reported for the dry-collected Evalyn HPV self-sampling brush (Rovers, Oss, The Netherlands) for up to 32 weeks at temperatures ranging from +4 °C to +30 °C [30], brush-based self-collected genital secretions stored in ThinPrep^®^ PreservCyt^®^ Solution (Hologic Bedford, MA, USA) at room temperature for 2 months or conserved on solid transport card at −80 °C [31], and self-collected vaginal swabs conserved in non-alcohol-based media for up to 4 weeks at +20–25 °C and +37 °C [32].

Our study has some limitations. Firstly, the number of tested samples is relatively low, and the kinetic of HPV DNA retrieval for molecular analysis after 27 months could not be carried out effectively. Secondly, we observed particularly high cumulative HPV loads in genital samples from FSWs. As this key population is at risk for several sexually transmitted infections, such high HPV loads would likely be due to the synergistic interactions between these infectious agents [39]. Such high HPV loads observed in our study could have introduced a recruitment bias of women with a higher genital load of HPV than what is observed in adult women from the general population. Thirdly, the Cyt-All^®^ UTM, which does not contain formaldehyde and methanol, thereby making it less toxic and easier to handle, is an alcohol-based preservative media, making it flammable and unsuitable for air transport. In our hands, vaginal samples were sent frozen to Europe, and the elution fluid was added to the laboratory, which requires additional costs for transport. A new, alcohol-free version of the Cyt-All^®^ medium is currently under development, which would make it possible to transport genital samples eluted in a preservative medium by air. Other alternative non-alcohol-based media could be used for vaginal self-sampling-based prevention programs for cervical cancer [32,40].

## 5. Conclusions

One major advantage of using the Cyt-All^®^ UTM lies in the fact that cervical samples could be safely stored at room temperature without the need for large infrastructures and resource costs. Veil-based self-collected genital secretions samples on UTM could then be a reliable and affordable approach to constitute large biobanks for HPV DNA molecular testing for screening for HPV infection in women with poor access to health care due to economic and cultural reasons. The use of the Cyt-All^®^ UTM for the conservation of anal samples collected by self-sampling could also be proposed for the screening of anal carcinoma in high-risk patients. Indeed, similarly to self-collected genital secretions for cervical cancer screening [41], even in sub-Saharan Africa [11,23] self-collected anal samples could be of interest to reach patients outside the health care system [42].

## Figures and Tables

**Table 1:** Detection using the BMRT Human Papillomavirus Genotyping Real-Time PCR Kit (Jiangsu Bioperfectus Technologies Co., Ltd.) of HPV genotypes retrieved from 30 HPV-positive veil-based self-collected genital samples embedded in the Cyt-All^®^ universal transport medium (Alphapath) and stored at either −30 °C (freezer), +4 °C (fridge), or at room temperature (+25 °C) for a 27-month period.

	ID	HPV Detection and Genotyping at Baseline (by Reference Assays) ^µ^	HPV Detection and Genotyping Using the BMRT Human Papillomavirus Genotyping Real-Time PCR Kit (Jiangsu Bioperfectus Technologies Co., Ltd.)		
			Storage at −30 °C	Storage at +4 °C	Storage at +25 °C
1	#A	HPV-35	HPV-35	HPV-35	HPV-35
2	#B	HPV-53; HP-V52	HPV-53; HPV-52	HPV-53; HPV-52	HPV-53; HPV-52
3	#C	HPV-66; HPV-58; HPV-45; **HPV-52 ^&^**	HPV-66; HPV-58; HPV-45	HPV-66; HPV-58; HPV-45	HPV-66; HPV-58; HPV-45
4	#D	HPV-53; HPV-52; HPV-6	HPV-53; HPV-52; HPV-6	HPV-53; HPV-52; HPV-6	HPV-53; HPV-52; HPV-6
5	#E	HPV-31; HPV-66; HPV-58; HPV-6	HPV-31; HPV-66; HPV-58; HPV-6	HPV-31; HPV-66; HPV-58; HPV-6	HPV-31; HPV-66; HPV-58; HPV-6
6	#F	HPV-58; HPV-45; HPV-56	HPV-58; HPV-45; HPV-56	HPV-58; HPV-45; HPV-56	HPV-58; HPV-45; HPV-56
7	#G	HPV-33; HPV-58; HPV-52; HPV-68; HPV-39	HPV-33; HPV-58; HPV-52; HPV-68; HPV-39	HPV-33; HPV-58; HPV-52; HPV-68; HPV-39	HP-V33; HPV-58; HPV-52; HPV-68
8	#H	HPV-53; HPV-52	HPV-53; HPV-52	HPV-53; HPV-52	HPV-53; HPV-52
9	#I	HPV-16; HPV-66; HPV-39	HPV-16; HPV-66; HPV-39	HPV-16; HPV-66; HPV-39	HPV-16; HPV-66; HPV-39
10	#J	HPV-52	HPV-52	HPV-52	HPV-52
11	#K	HPV-53; HPV-52	HPV-53; HPV-52	HPV-53; HPV-52	HPV-53; HPV-52
12	#L	HPV-53; HPV-52	HPV-53; HPV-52	HPV-53; HPV-52	HPV-53; HPV-52
13	#M	HPV-66; HPV-58; HPV-39; **HPV-53**	HPV-66; HPV-58; HPV-39	HPV-66; HPV-58; HPV-39	HPV-66; HPV-58; HPV-39
14	#N	HPV-53; HPV-52; HPV-6	HPV-53; HPV-52; **HPV-68**; HPV-6	HPV-53; HPV-52; **HPV-68**; HPV-6	HPV-53; HPV-52; **HPV-68**; HPV-6
15	#O	HPV-66; HPV-53; HPV-45; **HPV-58**; HPV-6	HPV-66; HPV-53; HPV-45; HPV-6	HPV-66; HPV-53; HPV-45; HPV-6	HPV-66; HPV-53; HPV-45; HPV-6
16	#P	HPV-66; HPV-53; HPV-33; HPV-58; HPV-6	HPV-66; HPV-53; HPV-33; HPV-58; **HPV-45**; HPV-6	HPV-66; HPV-53; HPV-33; HPV-58; **HPV-45**; HPV-6	HPV-66; HPV-53; HPV-33; HPV-58; **HPV-45**; HPV-6
17	#Q	HPV-16; HPV-18; HPV-31; HPV-45; HPV-52; HPV-35; HPV-68; HPV-11	HPV-16; HPV-18; HPV-31; HPV-45; HPV-52; HPV-35; HPV-68; HPV-11	HPV-16; HPV-18; HPV-31; HPV-45; HPV-52; HPV-35; HPV-68; HPV-11	HPV-16; HPV-18; HPV-31; HPV-45; HPV-52; HPV-35; HPV-68; HPV-11
18	#R	HPV-53; HPV-52; HPV-35; HPV-26	**HPV-16**; HPV-53; HPV-52; HPV-35; HPV-26	**HPV-16**; HPV-53; HPV-52; HPV-35; HPV-26	**HPV-16**; HPV-53; HPV-52; HPV-35; HPV-26
19	#S	HPV-33; **HPV-52**; HPV-51	HPV-33; **HPV-52**; HPV-51	HPV-33; HPV-51	HPV-33; HPV-51
20	#T	HPV-16; HPV-66	HPV-16; HPV-66	HPV-16; HPV-66	HPV-16; HPV-66
21	#U	HPV-66; HPV-53; HPV-52	HPV-66; HPV-53; HPV-52	HPV-66; HPV-53; HPV-52	HPV-66; HPV-53; HPV-52
22	#V	HPV-53; HPV-58; HPV-56	HPV-53; HPV-58; HPV-56	HPV-53; HPV-58; HPV-56	HPV-53; HPV-58; HPV-56
23	#W	HPV-31; HPV-53; **HPV-56**; HPV-52; **HPV-35**	HPV-31; HPV-53; **HPV-56**; HPV-52	HPV-31; HPV-53; **HPV-56**; HPV-52	HPV-31; HPV-53; HPV-52
24	#X	HPV-16; HPV-66; HPV-53; **HPV-59**	HPV-16; HPV-66; HPV-53	HPV-16; HPV-66; HPV-53	HPV-16; HPV-66; HPV-53
25	#Y	HPV-18; HPV-58; HPV-45; **HPV-82**	HPV-18; HPV-58; HPV-45	HPV-18; HPV-58; HPV-45	HPV-18; HPV-58; HPV-45
26	#Z	HPV-53; HPV-52	HPV-53; HPV-52	HPV-53; HPV-52	HPV-53; HPV-52
27	#Ø	**HPV-33**; HPV-58; HPV-45; HPV-68; HPV-51; HPV-39; HPV-82; HPV-81	**HPV-33**; HPV-58; HPV-45; HPV-68; HPV-51; HPV-39; HPV-82; HPV-81	HPV-58; HPV-45; HPV-68; HPV-51; HPV-39; HPV-82; HPV-81	HPV-58; HPV-45; HPV-51; HPV-39; HPV-82; HPV-81
28	#Ө	HPV-82; HPV-11	HPV-82; HPV-11	HPV-82; HPV-11	HPV-82; HPV-11
29	#Φ	HPV-59; HPV-66; HPV-33; HPV-58	HPV-59; HPV-66; HPV-33; HPV-58	HPV-59; HPV-66; HPV-33; HPV-58	HPV-59; HPV-66; HPV-33; HPV-58
30	#Ω	HPV-59; HPV-66	HPV-59; HPV-66	HPV-59; HPV-66	HPV-59; HPV-66

^µ^ Baseline HPV genotype detection at inclusion was carried out using Papilloplex^®^ High-Risk HPV and Papilloplex^®^ Low-Risk HPV (GeneFirst Ltd., Abingdon, UK); only the 21 HPV types detected using the BMRT Human Papillomavirus Genotyping Real-Time PCR Kit were analyzed. ^&^ HPV types differentially detected between the conditions analyzed are highlighted in bold.

**Table 2:** Two-by-two tables of any HPV and HR-HPV detection using the BMRT Human Papillomavirus Genotyping Real-Time PCR Kit (Jiangsu Bioperfectus Technologies Co., Ltd.) in 60 veil-based self-collected genital samples, including 30 samples positive at inclusion, which were determined using Papilloplex^®^ High-Risk HPV and Papilloplex^®^ Low-Risk HPV (GeneFirst Ltd.) as the reference molecular assays, and 30 HPV-negative control samples. Samples were stored in the Cyt-All^®^ universal transport medium (Alphapath) at −30 °C, at +4 °C, and at room temperature (+25 °C) for a 27-month period.

		Storage at −30 °C				Storage at +4 °C				Storage at +25 °C			
		BMRT HPV PCR				BMRT HPV PCR				BMRT HPV PCR			
		Any HPV		HR-HPV		Any HPV		HR-HPV		Any HPV		HR-HPV	
		Positive	Negative	Positive	Negative	Positive	Negative	Positive	Negative	Positive	Negative	Positive	Negative
**Reference HPV PCR**	**Positive**	100	3	64	2	98	5	62	4	95	8	59	7
	**Negative**	0	30	0	30	0	30	0	30	0	30	0	30
Sensitivity (%)		97.1		96.9		95.1		93.9		92.2		89.4	
Specificity (%)		100.0		100.0		100.0		100.0		100.0		100.0	
Agreement (%) ^a^		97.7		97.9		96.2		95.8		93.9		92.7	
Concordance (Cohen’s k coefficient) ^b^		0.94		0.95		0.90		0.91		0.84		0.84	
Accuracy (Youden’s J index) ^c^		97.1		96.9		95.1		93.9		92.2		89.4	

^a^ Agreement = TP + TN/TP + FP + TN + FN, expressed in percentage. ^b^ The Cohen’s κ coefficient calculation was used to estimate the concordance [20] and was interpreted according to the Landis and Koch scale [19] as follows: <0 indicates no agreement, 0–0.20 as slight, 0.21–0.40 as fair, 0.41–0.60 as moderate, 0.61–0.80 as substantial, and 0.81–1 as almost perfect concordance. ^c^ The accuracy of the storage conditions (Cyt-All^®^ universal transport medium and temperatures) associated with the use of the BMRT Human Papillomavirus Genotyping Real-Time PCR assay to correctly diagnose HPV infection was estimated using Youden’s J index (J = sensitivity+ specificity − 1) [22]. FN: false negative; FP: false positive; TN: true negative; TP: true positive.

## Data Availability

The data that support the conclusions of this study are available from the corresponding author upon reasonable request.
